# Endoplasmic Reticulum Stress Markers and Their Possible Implications in Leprosy's Pathogenesis

**DOI:** 10.1155/2018/7067961

**Published:** 2018-12-16

**Authors:** Kelly Emi Hirai, Jorge Rodrigues de Sousa, Luciana Mota Silva, Leônidas Braga Dias Junior, Ismari Perini Furlaneto, Francisca Regina Oliveira Carneiro, Tinara Leila de Souza Aarão, Mirian Nacagami Sotto, Juarez Antonio Simões Quaresma

**Affiliations:** ^1^Center of Biological and Health Sciences, State University of Pará, Belém, PA, Brazil; ^2^Tropical Medicine Center, Federal University of Pará, Belém, PA, Brazil; ^3^Evandro Chagas Institute, Ministry of Health, Ananindeua, PA, Brazil; ^4^School of Medicine, Sao Paulo University, Sao Paulo, SP, Brazil; ^5^Tropical Medicine Institute, Sao Paulo University, Sao Paulo, SP, Brazil

## Abstract

*Mycobacterium leprae* causes leprosy, a dermatoneurological disease which affects the skin and peripheral nerves. One of several cellular structures affected during *M. leprae* infection is the endoplasmic reticulum (ER). Infection by microorganisms can result in ER stress and lead to the accumulation of unfolded or poorly folded proteins. To restore homeostasis in the cell, the cell induces a series of signaling cascades known as the unfolded protein response called UPR (unfolded protein response). The present work is aimed at investigating the in situ expression of these markers in cutaneous lesions of clinical forms of leprosy and establish possible correlation expression patterns and types of lesion. A total of 43 samples from leprosy patients were analyzed by immunohistochemistry with monoclonal antibodies against GRP78/BiP, PERK, IRE1*α*, and ATF6. A statistically significant difference between the indeterminate, tuberculoid, and lepromatous clinical forms was detected, with high expression of GRP78/BiP, PERK, IRE1*α*, and ATF6 in tuberculoid forms (TT) when compared to lepromatous leprosy (LL) and indeterminate (I) leprosy. These results represent the first evidence of ER stress in samples of skin lesions from leprosy patients. We believe that they will provide better understanding of the complex pathogenesis of the disease and facilitate further characterization of the cascade of molecular events elicited during infection.

## 1. Introduction

Leprosy is the clinical manifestation of a dermatoneurological disease caused by infection with *Mycobacterium leprae*. The interaction between *M. leprae* and the host is complex, and the disease presents a chronic evolution that affects mainly the skin and peripheral nerves. Its clinical manifestations vary and are associated with diverse host-dependent factors such as the pattern of innate and adaptive immune response, as well as genetic and immunogenetic factors [1–3]. Immune response patterns to *M. leprae* have been shown to involve components of innate immunity, such as dendritic cells, macrophages (both M1 and M2 subtypes), and natural killer cells, as well as diverse types of lymphocytes, such as T helper cells (Th1, Th2, Th17, Th9, Th22, and Th25) [4–8].

The bacterium-cell interaction activates various cellular response pathways responsible for recognizing the microorganism, resisting virulence factors, or triggering an elimination response [9–11]. In situ techniques have revealed alterations in several cellular structures and signaling pathways in the skin during such response. One of them is the endoplasmic reticulum (ER), which is involved in the biosynthesis of lipids and proteins, as well as intracellular signaling, and is, therefore, essential for the proper functioning of the cell [12–14].

Infection by microorganisms can result in ER stress, leading to the accumulation of unfolded or poorly folded proteins. To restore homeostasis in the ER, the cell induces a series of signaling cascades known as unfolded protein response (UPR). The UPR depends on three resident sensors: inositol-requiring transmembrane kinase/endoribonuclease 1 (IRE1), protein kinase R- (PKR-) like endoplasmic reticulum kinase (PERK), and activating transcription factor-6 (ATF6) [15, 16]. In the absence of stress, the UPR signaling pathways remain inactive. These pathways are linked to glucose-regulated protein 78 (GRP78). This chaperone has two main functions: (i) transfer unfolded proteins to the cytoplasm and aid in the process of ubiquitination and degradation and (ii) accelerate the ATP-mediated protein folding process with transfer to the Golgi complex [16–19]. Once ER stress is detected, GRP78 dissociates from IRE1, PERK, and ATF6, initiating the signaling cascade responsible for restoring equilibrium in the ER. However, when attempts to restore homeostasis fail, the programmed cell death process begins [20–25].

Studies have shown the influence of ER stress during bacterial infection. *M. tuberculosis* possesses a 38 kDa antigen that increases the expression of a proinflammatory cytokine, MCP-1-induced protein (MCPIP), which can generate reactive oxygen species (ROS) and cause the accumulation of ER proteins [26, 27]. Lim et al. observed that ER stress was significantly increased in M1 macrophages, which then efficiently removed intracellular *M. tuberculosis*. Hence, ER stress may be an important element in the host's immune response against bacteria in M1 macrophages [28].

Other studies have demonstrated a correlation between signaling pathways against unfolded proteins and the induction of gastric carcinogenesis caused by *Helicobacter pylori* infection. This correlation is mediated by the action of the vacuolizing cytotoxin (VacA) on gastric epithelial tissue cells. VacA intoxication and PERK activation result in the induction of C/EBP homologous protein (CHOP, also known as GADD153), apoptosis, and mitochondrial dysfunction [29, 30]. Shima et al. observed that the ER was in direct contact with the inclusions of *Chlamydia pneumoniae* induced by interferon gamma (IFN-*γ*) and that GRP78/BiP was induced during the early phase of infection. Increased GRP78/BiP expression was accompanied by the phosphorylation of eukaryotic initiation factor 2 alpha (eIF2a) [31]. This finding led researchers to conclude that increased chaperone expression attenuated ER stress-mediated apoptosis, suggesting that the activation of eIF2*α* and the induction of GRP78/BiP are important to revert ER stress conditions following persistent IFN-*γ*-eliciting infection. Methicillin-resistant *Staphylococcus aureus* (MRSA) was used as a model to prove how ER stress promoted antimicrobial functions [31]. Abuaita et al. observed that MRSA infection activated IRE1*α*, the most conserved protein in the UPR, which, in turn, promoted the generation of reactive oxygen species (ROS) as a safeguard mechanism against those bacterial pathogens that might have evaded the initial oxidative burst of macrophages [32].

Several reports, including on *M. tuberculosis*, have demonstrated the importance of alterations to the ER during an antimicrobial response; however, no such studies have looked at infection by *M. leprae* [26, 27, 32]. The objective of the present work was to investigate the in situ expression of ER stress markers in cutaneous lesions from leprosy patients and correlate them with the clinical forms studied.

## 2. Materials and Methods

### 2.1. Study Design

A total of 43 untreated patients with a confirmed diagnosis of leprosy according to the criteria recommended by the Madrid classification (1953) [33] were selected from the Dermatology Service of the State University of Pará between the years 2013 and 2017. Of these, 13 presented indeterminate leprosy (IL), 15 presented tuberculoid leprosy (TT), and 15 presented lepromatous leprosy (LL).

All clinical investigation has been conducted according to the principles expressed in the Declaration of Helsinki and Resolution N^o^ 466/2012 of the National Health Council of Brazil. After a complete description and explanation of the study design, written informed consent was obtained from all participants. The study was approved by the Ethics Committee of Tropical Medicine Center, Federal University of Pará, Brazil (protocol number 1.811.566).

### 2.2. Histopathology and Immunohistochemistry

For histopathological analysis, histological sections of tissue biopsies with a thickness of 5 *μ*m embedded in paraffin were stained by hematoxylin-eosin, Ziehl-Neelsen staining, and subsequent tissue immunostaining with specific monoclonal antibodies.

Immunohistochemistry with monoclonal antibodies (all Abcam at 1 : 100 dilution) against the phosphorylated form of GRP78/BiP (ab108613), PERK (ab79483), IRE1*α* (ab42187), and ATF6 (ab135707) was based on the formation of a biotin-streptavidin peroxidase complex as described by Quaresma et al. [34]. Tissue samples were first dewaxed in xylol and hydrated in ethyl alcohol. Then, endogenous peroxidase was blocked with 3% H_2_O_2_ for 45 min. After antigen retrieval with citrate buffer (pH 6.0) for 20 min at 90°C, nonspecific proteins were blocked with 10% concentrated skim milk for 30 min. Next, histological sections were incubated with diluted primary antibodies and 1% bovine serum albumin for 14 h, immersed in 1x phosphate-buffered saline (PBS), and then incubated with a biotinylated secondary antibody (LSAB kit; DakoCytomation) at 37°C for 30 min. The slides were then immersed again in 1x PBS and incubated with streptavidin peroxidase (LSAB kit) at 37°C for 30 min. After this interval, cuts were revealed following application of a chromogen solution (0.03% diaminobenzidine and 3% H_2_O_2_), stained with Harris hematoxylin for 1 min, dehydrated in ethyl alcohol, and cleared in xylol.

### 2.3. Quantitative Analysis

Immunostaining was quantified based on five visual fields randomly selected using a graduated grid with 10 × 10 subdivisions and 0.0625 mm^2^ area, as observed with a Zeiss Axio Imager Z1 microscope (400x).

### 2.4. Statistical Analysis

Results were tabulated using Excel® 2016 (Microsoft Corp.), and statistical analysis was performed with GraphPad Prism 5.0 (GraphPad Inc.). Frequencies, measures of central tendency, and dispersion were obtained during univariate analysis. ANOVA, Tukey's test, and Pearson's correlation coefficient were applied to investigate the experimental hypothesis. All tests were performed using a significance level of 5% (*p* ≤ 0.05).

## 3. Results

### 3.1. Clinical Aspects

Individuals included in the present study were from the eastern Brazilian Amazon, State of Pará, Brazil. They presented clinical features characterized by alterations in tactile, thermal and/or pain sensitivity, and cutaneous lesions. These consisted of imprecise hypochromic spots, sometimes hypoesthetic in I, erythematous or erythematous-hypochromic plaques with sharp edges and usually anesthetic in TT, and diffuse, erythematous-violet or erythematous plaques, infiltrated, bright, and sometimes coalescing in LL.

### 3.2. Histopathology

**Figure 1:**
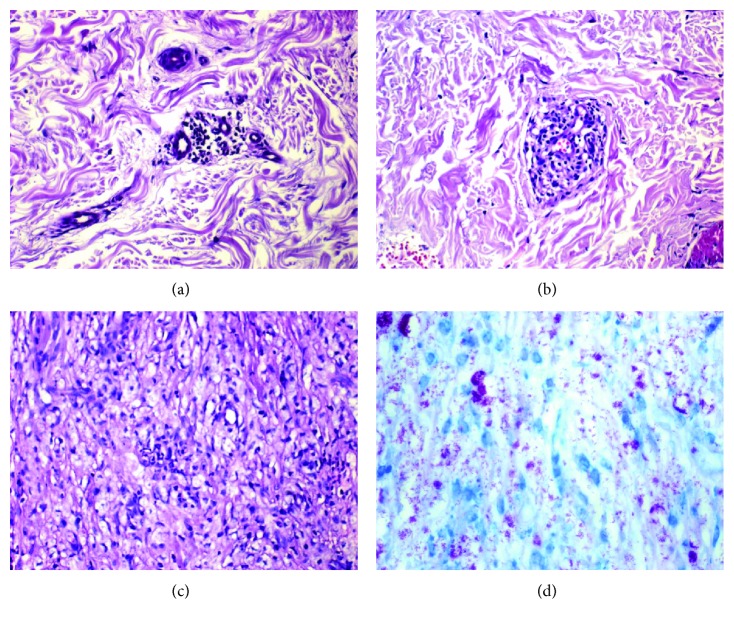
Histopathological aspects of leprosy lesions in I, TT, and LL clinical forms showing mild to severe inflammatory lymphohistiocytic infiltrate and the bacillus stained by the method of Ziehl-Neelsen (200x). (a) Indeterminate leprosy; (b) tuberculoid leprosy; (c) lepromatous leprosy; (d) Ziehl-Neelsen staining.

The histopathological characteristics of the lesions were visible in the I focal lymphohistiocytic inflammatory infiltrate distributed around appendages, nervous fillets, and vessels; sometimes, they were positive by bacilloscopy. In TT lesions, granulomas consisted of clustered epithelioid cells, sometimes surrounded by a dense or mild lymphocytic halo, with bacillus-negative tissue. In LL lesions, a granulomatous infiltrate consisting of histiocytes and plasma cells was detected, extending along the entire upper dermis and surrounding nerves and blood vessels. Such extension of the infiltrate might compromise the deep dermis until the hypodermis, thus preventing macrophages from eliminating the bacilli and allowing them instead to accumulate in the cytoplasm, sometimes in globies as demonstrated by Ziehl-Neelsen staining (Figure 1).

### 3.3. Immunohistochemistry

Immunostaining revealed areas of brownish color in the cytoplasm when tissues were probed with antibodies against all markers and in the nucleus in the case of ATF6 and PERK.

**Figure 2:**
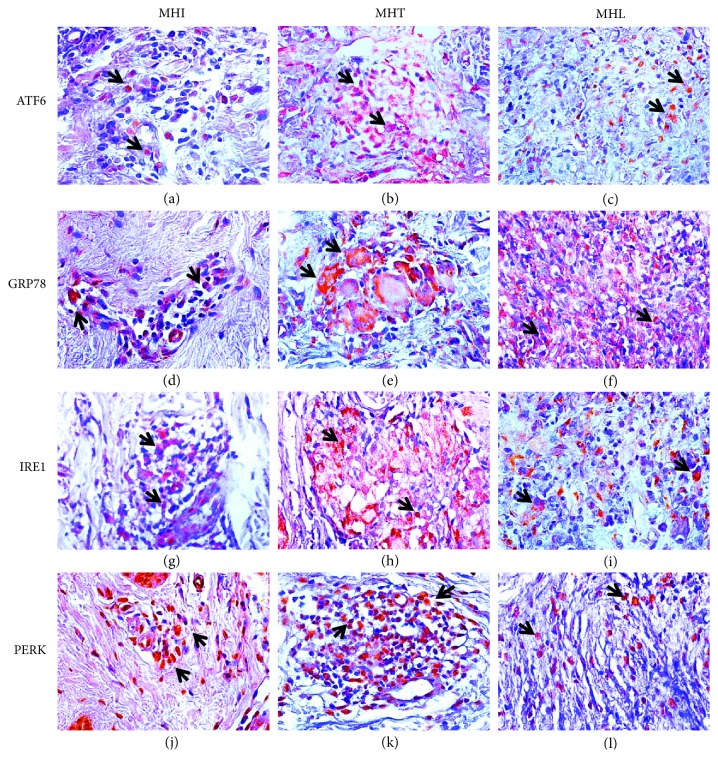
Immunohistochemistry for the ER stress markers in clinical forms I, TT, and LL of leprosy. Note that positive labeling is characterized by a brownish cytoplasmic pattern on granuloma cells (400x). (d) Indeterminate leprosy, GRP78/BiP antibody; (e) tuberculoid leprosy, GRP78/BiP antibody; (f) lepromatous leprosy, GRP78/BiP antibody; (j) indeterminate leprosy, PERK antibody; (k) tuberculoid leprosy, PERK antibody; (l) lepromatous leprosy, PERK antibody; (g) indeterminate leprosy, IRE1*α* antibody; (h) tuberculoid leprosy, IRE1*α* antibody; (i) lepromatous leprosy, IRE1*α* antibody; (a) indeterminate leprosy, ATF6 antibody; (b) tuberculoid leprosy, ATF6 antibody; (c) lepromatous leprosy, ATF6 antibody.

**Figure 3:**
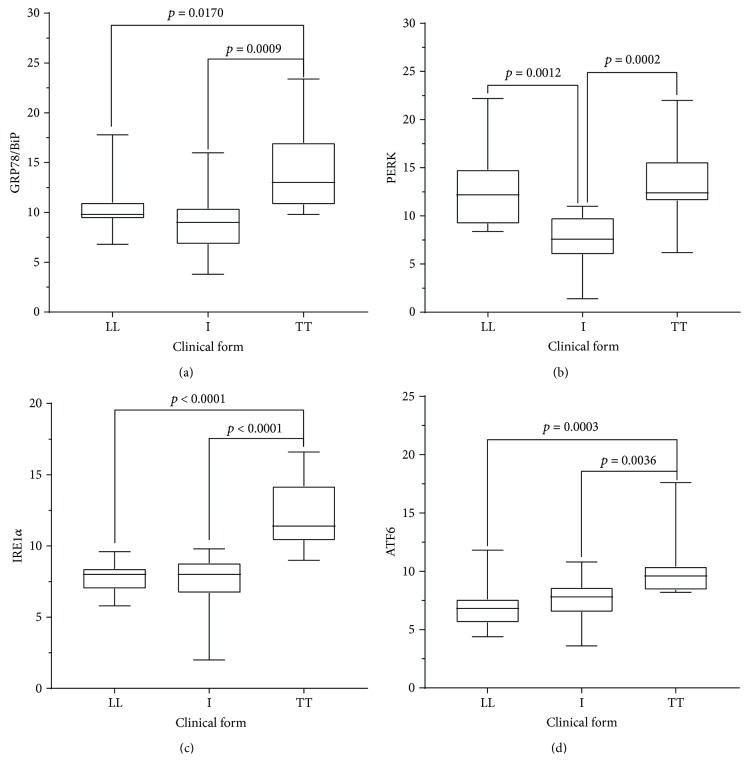
Mean and standard deviation of positive cell counts for ER stress markers in clinical forms of leprosy. Note that high expression was observed in TT clinical forms.

Immunostaining quantification indicated a statistically significant difference between the I, TT, and LL clinical forms. Specifically, cell expression of GRP78/BiP was statistically higher in TT lesions (13.84 ± 3.9) than in LL lesions (10.41 ± 2.57) or in I lesions (8.96 ± 3.15). PERK expression was also higher in TT lesions (13.33 ± 3.68) than in LL (12.48 ± 3.77) or IL (7.49 ± 2.61) lesions; however, the difference was statistically significant only between TT and IL and LL and IL clinical forms, but not between TT and LL lesions. IRE1*α* was highly expressed in TT (12.21 ± 2.35), followed by LL (7.84 ± 1.14), and I (7.34 ± 2.17) lesions, with a statistically significant difference between TT and the other clinical forms (Figures 2 and 3 and Table 1). ATF6 was more expressed in TT (10.11 ± 2.38), followed by I (7.48 ± 1.77), and LL (6.93 ± 1.77) clinical forms, with statistical difference between TT and the other clinical presentations of leprosy.

**Figure 4:**
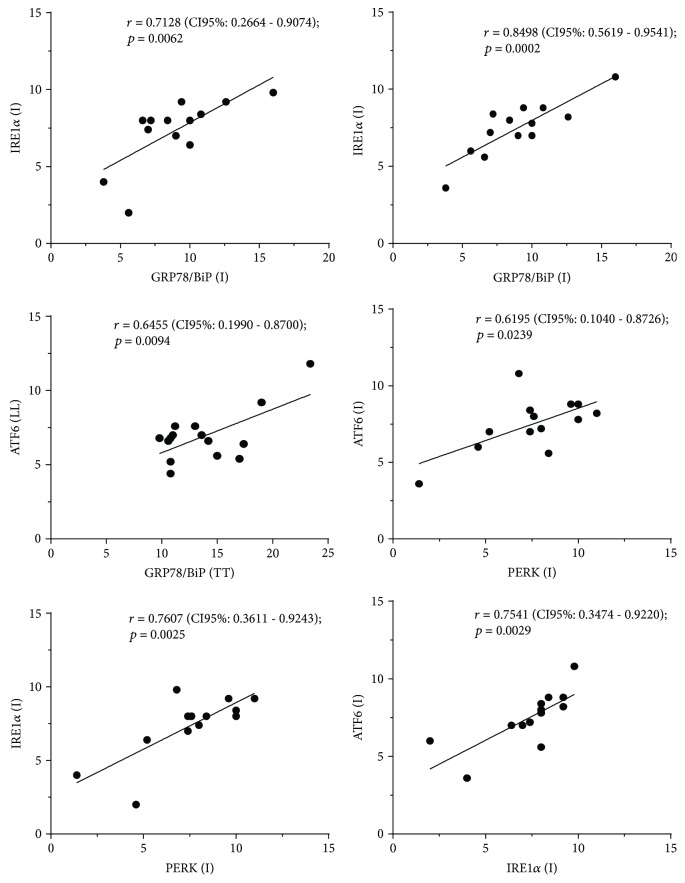
Statistically significant correlations between ER stress markers in I, TT, and LL clinical forms of leprosy.

According to parametric analysis, statistically significant values and a positive correlation were found for IRE1*α* and GRP78/BiP (*r* = 0.7128; *p* = 0.0062), IRE1*α* and PERK (*r* = 0.7607, *p* = 0.0025), ATF6 and PERK (*r* = 0.6195; *p* = 0.0239), ATF6 and GRP78/BiP (*r* = 0.8498; *p* = 0.0002), and ATF6 and IRE1*α* (*r* = 0.7541, *p* = 0.0029) in the I form. A positive correlation was found also between the LL form for ATF6 and TT form for GRP78/BiP (*r* = 0.6455; *p* = 0.0094) (Figure 4 and Table 2).

## 4. Discussion

Leprosy is a serious public health problem in several countries, and, despite being a long-known disease, its pathogenicity remains complex and still subject to clinical, epidemiological, molecular, and immunological studies. The interaction of *M. leprae* with host cells is based on virulence and evasion of the infectious agent, as well as recognition and elimination of the agent by the host's immune response [3–7]. Many of these relationships depend on the way *M. leprae* interacts with target cells, particularly macrophages, and how the various subpopulations of macrophages trigger the mechanisms of bacillus recognition and elimination. During this interaction, mechanisms related to phagosome formation, phagolysosome activity, production of nitric oxide (NO) and ROS, changes in lipid metabolism, expression of cytokines, and other intracellular processes are triggered by the presence of *M. leprae* within macrophages [5, 35, 36]. Several infectious agents have been shown to induce ER stress, including dengue virus, Zika virus, hepatitis B and C virus, and enterovirus, as well as bacteria, such as *M. tuberculosis*, *Staphylococcus*, *H. pylori*, and fungi of the *Candida* genus. Some studies have pointed to the direct influence of ER stress on the host's response and ability to eliminate infectious pathogens [26, 27, 32, 37–43].

Our findings reveal an increase in the expression of factors involved in the cellular UPR. Lim et al. correlated the occurrence of ER stress to subpopulations of M1 macrophages in tuberculosis, but not M2 macrophages [27, 28, 35]. Our data corroborate these findings, as expression of ER stress response markers was higher in TT lesions, where M1 macrophages are predominant, whereas M2 macrophages are related to LL lesions [28]. Specifically, GRP78/BiP, IRE1*α*, PERK, and ATF6 were more expressed in TT lesions than in I and LL lesions. M1 macrophages can release ROS and NO, as well as proinflammatory cytokines such as tumor necrosis factor alpha (TNF-*α*) and interleukin- (IL-) 6, which may explain the association with ER stress. The detection of these proinflammatory cytokines or Th1 cells mainly in TT lesions or in leprosy reactions corroborates our findings [7].

*In vivo* and *in vitro* models have shown that apoptosis is an important mechanism of cell death related to mycobacterial infections, including leprosy [11, 36, 44]. ER stress can induce cell death by apoptosis, facilitating the elimination of mycobacteria in M1 macrophages during tuberculosis via activation of the Toll-like receptor (TLR) 2 intracellular signaling pathway [27, 28]. Cell death may have antagonistic effects on the relationship between *M. tuberculosis* and host cells, as necrosis favors proliferation of the bacillus, whereas apoptosis favors infection control [26, 28]. In addition, autophagy may be caused by the immune mechanisms triggered by cytokines and intracellular enzymes [28]. Thus, as already demonstrated in previous studies, the mechanism of cell death participates effectively in the control of leprosy infection and, depending on the clinical form, may contribute to evasion of the host's immune responses or even cooperate towards the most effective microbicidal response [11, 36, 44]. Watson et al. demonstrated that the presence of *M. tuberculosis* in the cytosol could induce type I interferon via activation of the stimulator of interferon gene (STING) cascade and thus promote autophagy [45]. As STING resides in the ER, infection by virulent or attenuated strains of *M. tuberculosis* may lead to alteration of ER homeostasis. Here, ER stress markers were expressed in LL clinical forms, which could reflect a response to a greater presence of bacilli in these lesions. As in tuberculosis, apoptosis plays a key role in the formation of granuloma against *M. leprae* infection in the TT and LL clinical forms of the disease [27, 28].

GRP78/BiP presented a differential expression among the clinical forms studied, with increased levels in TT. These data point to the probable involvement of *M. leprae* in ER stress induction, as observed in tuberculosis. GRP78/BiP is expressed in response to stressful stimuli, such as glycopenia, decreased oxygen, and low Ca^2+^ concentration. It is abundant in the ER and mitochondria, where it could protect infected macrophages from apoptosis induced by mitochondrial dysfunction and cytochrome c release [16–19].

Expression of IRE1*α* was more intense in the TT form than in the LL or I clinical forms. After dissociation of GRP78, the cytosolic domain of IRE1*α* (15) undergoes autophosphorylation and the RNAse domain processes an intron of X box-binding protein-1 (XBP-1) mRNA to allow the production of XBP-1. The latter acts as a transcription factor that promotes expression of chaperones such as GRP78 and maintains ER homeostasis [18, 25]. Macrophages infected with *M. tuberculosis* and undergoing ER stress trigger XR-1-dependent TLR hyperregulation, an important mechanism of infection control. In addition, the presence of *M. tuberculosis* antigens can stimulate apoptosis via activation of the IRE1*α*/TNF receptor-associated factor 2 (TRAF2)/mitogen-activated protein kinase kinase kinase 5 (ASK1) pathway, whereas IRE1*α* regulates the production of microRNAs, leading to caspase expression. The increased expression of IRE1*α* observed in the TT leprosy form suggests its participation in the cellular response to *M. leprae*, probably via mechanisms akin to those observed for *M. tuberculosis* [27, 46–48].

PERK expression was higher in TT lesions compared to other clinical forms; however, any statistical difference was observed only between the TT/LL and the I clinical forms. During ER stress, PERK initiates a decrease in overall protein synthesis by increasing eIF2*α* phosphorylation. This switch depends on promoting translation of the ATF4 transcription factor responsible for inducing the expression of genes involved in the activation of the UPR pathway [22, 48, 49]. If the stress is prolonged or not reversed, ATF4 mediates the expression of genes that contribute to apoptosis through the induction of numerous proapoptotic proteins, including protein phosphatase 1 regulatory subunit 15A (GADD34) [24].

Tissue expression of ATF6 followed the same trend as those of previously described markers, with increased expression in the TT lesion. After dissociation, ATF6 is transferred to the Golgi complex where it is cleaved by site-1 (S1P) and S2P proteases and then translocated to the nucleus as an activated transcription factor. There, it binds to stress response elements of the ER to activate target genes, such as GRP94 and CHOP (9-11). ATF6 can also regulate the expression of and bind to XBP-1 protein, thus promoting the UPR [20–22].

The expression of all examined markers was low in the early I clinical form of the disease. Even though its symptoms are not always characteristic and are sometimes associated with neural lesions that culminate in altered sensitivity, the I form has histopathological and immunohistochemical features characterized by discrete inflammatory infiltrates commonly present in the initial phase of the infection/disease [50]. This absence of a more abundant lymphohistiocytic infiltrate is perhaps the main factor explaining why ER stress response markers are only weakly expressed. As the infection evolves, they can increase in association with the pattern of immune response related to a characteristic clinical form of the diseases [50].

Finally, in an overview, the relationship of ER stress with leprosy shows that the participation of these markers seems to be key to the understanding of the immunopathogenesis of the disease by the fact that the regulatory effect of the UPR pathway can have direct repercussion in the activation of genes that modulate apoptosis and the immune response of the host. Interestingly, because clinical forms are studied in this work, where the in situ immune response pattern is different, the activation of the UPR pathway probably can be a determinant for the control of bacillary proliferation or the microbicidal response. Therefore, the tissue damage observed due to the polarization of response between M1 and M2 macrophages triggers an antagonistic effect where it is believed that in the LL form, apoptosis serves as a strategy of immune evasion to deregulate immune surveillance. In this context, the response of cytokines such as TGF-*β* while enhancing the cell death process also promotes tissue repair. In this scenario, the ER stress appears to be relevant mainly to the heterogeneous response of the host. Because the specific immune response in the TT form is more effective, the ER stress in this case may influence the production of proinflammatory cytokines such as TNF-*α* and IFN-*γ*, which in addition to activating M1 macrophages, causes the generation of ROS to destroy the bacillus. In an indeterminate way, the effect resulting from the activation of the UPR pathway compared to the other clinical forms indicates that the ER stress is less accentuated to the point that as it deals with the primary evolution of the disease, the immune response corroborates with the presence of an inflammatory infiltrate where tissue damage is less evidenced compared to the TT and LL forms of the disease.

## 5. Conclusion

The data presented here represent the first report of ER stress in samples of skin lesions from leprosy patients and will improve our understanding of the complex pathogenesis of the disease. The results observed in this work corroborate the findings described in other mycobacterial infections such as tuberculosis where the expression of ER stress markers is associated with proinflammatory immune responses. These data open possibilities of future investigations correlating the occurrence of deformities observed in leprosy with the induction of higher ER stress and cell death, mainly observed in TT forms where these markers were most expressed. However, it is worth emphasizing that these findings are of particular benefit for the detailed characterization of the cascade of molecular events involved in the cell-pathogen or host-pathogen interaction during *M. leprae* infection in its various clinical forms and reaction conditions.

## Figures and Tables

**Table 1:** Quantitative analysis of GRP78/BiP, PERK, IRE1*α*, and ATF6 levels according to the clinical form of leprosy.

Markers	Clinical form of leprosy			*p* value^∗^
	I Mean ± SD (95% CI)	TT Mean ± SD (95% CI)	LL Mean ± SD (95% CI)	
GRP78/BiP	8.96 ± 3.15 (7.05–10.86)	13.84 ± 3.90 (11.68–16.00)	10.41 ± 2.57 (8.99–11.84)	0.0009^†^
PERK	7.49 ± 2.61 (5.92–9.07)	13.33 ± 3.68 (11.30–15.37)	12.48 ± 3.77 (10.39–14.57)	0.0001^†^
IRE1*α*	7.34 ± 2.17 (6.03–8.65)	12.21 ± 2.35 (10.91–13.51)	7.84 ± 1.14 (7.21–8.47)	<0.0001^†^
ATF6	7.48 ± 1.77 (6.41–8.55)	10.11 ± 2.38 (8.79–11.42)	6.93 ± 1.77 (5.96–7.91)	0.0002^†^

I: indeterminate; LL: lepromatous; TT: tuberculoid; 95% CI: 95% confidence interval; SD: standard deviation. ^∗^Ordinary one-way ANOVA (*p* < 0.05). ^†^Statistically significant.

**Table 2:** Correlation coefficient of the ER stress markers in TT, LL and I leprosy clinical forms.

Markers and clinical forms		GRP78/BiP			PERK			IRE1*α*			ATF6	
		LL	I	TT	LL	I	TT	LL	I	TT	LL	I
GRP78/BiP	I	0.3219										
	TT	0.3069	−0.0715									
PERK	LL	0.1500	0.0515	0.3420								
	I	0.2173	0.5246	0.4124	−0.1249							
	TT	0.5477	−0.0776	0.4657	0.5106	−0.0220						
IRE1*α*	LL	−0.1010	−0.0102	0.3854	0.2044	−0.1069	0.1716					
	I	0.0093	0.7128^∗∗^	0.1599	−0.1701	0.7607^∗∗^	−0.0214	0.0908				
	TT	0.1138	0.3982	−0.3297	−0.0851	0.3591	−0.1802	−0.1331	0.4021			
ATF6	LL	0.1738	−0.4045	0.6455^∗∗^	−0.1054	0.1600	0.1843	0.4588	0.0009	−0.0335		
	I	0.2522	0.8498^∗∗^	−0.1206	−0.2736	0.6195^∗^	−0.1524	−0.1654	0.7541^∗∗^	0.4547	−0.3302	
	TT	−0.0452	−0.2149	−0.0335	−0.1024	−0.0974	−0.1919	0.2342	−0.3351	0.3320	0.2078	−0.3466

LL: lepromatous; I: indeterminate; TT: tuberculoid. Correlation matrix. ^∗^*p* < 0.05; ^∗∗^*p* < 0.01.

## Data Availability

The datasets used and/or analyzed during the current study are available from the corresponding author on reasonable request.
